# Prevalence and determinants of domain specific cognitive impairment in older adults in North India

**DOI:** 10.1590/1980-5764-DN-2025-0396

**Published:** 2026-08-21

**Authors:** Shubhanjali Roy, Shomik Ray

**Affiliations:** 1 Public Health Foundation of India, Indian Institute of Public Health-Delhi, Department of Health Technology Assessment, Gurugram, Haryana, India.

**Keywords:** Cognitive Dysfunction, Aged, Self Care, Healthy Ageing, Geriatrics, Urban Health Services, Disfunção Cognitiva, Idoso, Autocuidado, Envelhecimento Saudável, Geriatria, Serviços Urbanos de Saúde

## Abstract

The rapid growth of India's elderly population necessitates understanding the determinants of cognitive health.

**Objective:**

This study examined the prevalence of cognitive impairment and its association with socio-demographic, health, and behavioural factors among community-dwelling older adults in urban Delhi.

**Methods:**

A community-based cross-sectional study was conducted from January to June 2023 among 290 adults aged ≥60 years in South-West Delhi, selected through multistage sampling. Data on socio-demographics, health status, and lifestyle were collected via structured interviews. Cognitive function was assessed using the Brief Interview for Mental Status (BIMS) and classified as intact, mild to moderate, or severe impairment. Principal Component Analysis (PCA) identified explanatory dimensions; multivariable ordinal logistic regression examined associations; and K-means clustering grouped participants by cognitive profiles.

**Results:**

Overall, 52.8% had intact cognition, 37.2% mild to moderate impairment, and 10% severe impairment. Severe impairment was most prevalent among those aged ≥81 years (61.5%) and with no formal education (55.2%), while intact cognition was highest among skilled workers (26.6%) and those with high school or higher education (43.8%). Significant factors included age, education, body mass index (BMI), physical activity, smoking, alcohol use, dietary intake, sleep quality, mental activity, and social engagement (all p<0.001). PCA yielded eight principal components capturing behavioral, health, and socio-demographic domains. K-means clustering identified two distinct cognitive profiles based on performance and associated factors.

**Conclusion:**

Cognitive status in older urban adults is shaped by modifiable health and lifestyle factors. Community-based interventions promoting physical activity, social participation, and healthy behaviors may help preserve cognition in aging populations.

## RESUMO

O rápido crescimento da população idosa na Índia exige a compreensão dos determinantes da saúde cognitiva.

**Objetivo:**

Este estudo examinou a prevalência de comprometimento cognitivo e sua associação com fatores sociodemográficos, de saúde e comportamentais entre idosos que vivem em comunidade na área urbana de Délhi.

**Métodos:**

Um estudo transversal baseado na comunidade foi realizado de janeiro a junho de 2023, com 290 adultos com idade ≥60 anos, no sudoeste de Deli, selecionados por meio de amostragem em várias etapas. Dados sociodemográficos, de estado de saúde e estilo de vida foram coletados por meio de entrevistas estruturadas. A função cognitiva foi avaliada usando a Entrevista Breve para o Estado Mental (BIMS) e classificada como intacta, leve ou grave. A Análise de Componentes Principais (PCA) identificou dimensões explicativas; a regressão logística ordinal multivariável examinou associações; e o agrupamento K-means agrupou os participantes por perfis cognitivos.

**Resultados:**

No geral, 52,8% tinham cognição intacta, 37,2% comprometimento leve e 10% comprometimento grave. O comprometimento grave foi mais prevalente entre aqueles com idade ≥81 anos (61,5%) e sem educação formal (55,2%), enquanto a cognição intacta foi mais alta entre trabalhadores qualificados (26,6%) e aqueles com ensino médio ou superior (43,8%). Os fatores significativos incluíram idade, educação, índice de massa corporal (IMC), atividade física, tabagismo, consumo de álcool, ingestão alimentar, qualidade do sono, atividade mental e envolvimento social (todos p<0,001). A PCA produziu oito componentes principais que capturaram os domínios comportamental, de saúde e sociodemográfico. O agrupamento K-means identificou dois perfis cognitivos distintos com base no desempenho e nos fatores associados.

**Conclusão:**

O estado cognitivo em idosos urbanos é moldado por fatores modificáveis de saúde e estilo de vida. Intervenções comunitárias que promovam a atividade física, a participação social e comportamentos saudáveis podem ajudar a preservar a cognição em populações envelhecidas.

## INTRODUCTION

The population of people 65 and older is increasing more rapidly than the population of people of all other ages worldwide. According to projections from the United Nations’ (UN) 2019 report on World Population Prospects, one in six people (or 16%) will be over 65 in 2050, up from one in eleven (9%) in 2019. In Europe and North America, one in four people could be 65 or older by 2050. For the first time ever in 2018, adults 65 and older outnumbered youngsters under five around the world. By 2050, there will be 426 million people who are 80 years of age or older, which is expected to quadruple the current population of 143 million^1^. The UN Decade of Healthy Ageing (2021–2030) aims to reduce health inequities and improve the lives of older people, their families, and communities by taking collective action in four areas: changing our attitudes towards ageing and ageism; building communities that support older people's abilities; and offering person-centered integrated care and primary health services responsive to older people^2^. India currently has the second-largest elderly population in the world, with 140 million residents 60 and older. Additionally, the senior population is expanding at a rate that is about three times faster than India's overall population growth rate^3,4^. The elderly population increased by around 27 million people between 2001 and 2011. In 2011–21 and 2021–31, respectively, this rise is projected to be 34 million and 56 million. According to the report from the 2020 National Commission on Population, there would be 67 million men and 71 million women aged 65 and older in India by 2021^5^. According to the 2011 census, there were 103 million senior people in India, or 8.6% of the country's total population^6^.

The modern social structure, which is shifting from the extended joint family system to nuclear families (defined as parents and their dependent children), as well as migration to urban areas and other countries, will put a great deal of strain on the current system, necessitating proactive government interventions^7,8^. The elderly population is more susceptible to many health disorders as a direct consequence of the natural ageing process and other social determinants^9^. Elderly people are likely to have cardiovascular disorders such a hypertension, coronary artery disease, heart failure, and stroke^10^. Additionally, more prevalent age-related health issues include osteoarthritis, which leads to inflammation and reduced joint movement, osteoporosis, which can cause bone fragility and weakness that can make fractures more frequent^11^, and Alzheimer's disease, a type of dementia that impairs memory and cognitive function. Type 2 diabetes is more prevalent and, if not properly controlled, can cause complications. Chronic obstructive pulmonary disease (COPD), asthma, and pneumonia are respiratory conditions that can cause difficulty in breathing^12^. As people age, their risk of developing different types of cancer also rises. Furthermore, age-related macular degeneration, cataracts, glaucoma, and hearing loss are among conditions that can impair sensory experience^13^.

Emotional and social loneliness can be due to life changes, loss of loved ones, social isolation, and health problems, which can all cause depression in elderly people^14,15^. Falls and fractures are more likely as a result of poor coordination, muscle weakness, and balance that could be associated with various chronic diseases at this age^16^. The most prevalent mental and neurological disorders are dementia and depression, which affect roughly 5 and 7% of the world's older population, respectively. Studies suggest that over 20% of elderly aged 60 and above suffer from a mental or neurological disorder (excluding headaches), and 7% of all disabilities among elderly persons over 60 are attributed to mental and neurological disorders^17^. One of the related conditions among elderly population is cognitive impairment. According to the recent studies, the global prevalence of cognitive impairment among the elderly is estimated from 5.1 to 41.1%^18^. A decline in cognitive abilities, such as visuospatial skills, attention, memory, thinking, reasoning, and problem-solving ability, is referred to as cognitive impairment. Senior citizens frequently worry about their health because of it. From moderate cognitive impairment, which is a perceptible reduction in cognitive function but is not severe enough to interfere with daily activities, to more serious illnesses like dementia, including Alzheimer's disease, cognitive impairment can take many different forms^19^. However, it is also normal for people to experience certain cognitive changes as they get older. A person's cognition has a significant association with the socio-demographic determinants, like education, wealth status, nutrition, and occupation^20^. However, severe cognitive impairment can have a major effect on older individuals’ quality of life and independence in performing their daily duties. Their interpersonal interactions, emotional health, and general functioning may also be impacted. This study investigates how social, behavioral and self-care-related factors — particularly low physical activity, smoking, alcohol consumption, family and social interactions and other pathophysiological or comorbidities — are associated with an increased risk of cognitive impairment, defined as reduced performance across memory, orientation, and attention domains, and classified into intact, mild to moderate, and severe levels. It further examines whether the frequency and duration of these social, behavioral and self-care-related determinants, along with co-existing chronic conditions, contribute to the greater severity of cognitive decline.

This study aims to assess domain-wise cognitive profiles among community-dwelling older adults in urban Delhi using the Brief Interview for Mental Status (BIMS). It further seeks to estimate the prevalence of cognitive impairment and examine social, behavioral, sociodemographic, and psychosocial determinants — including both modifiable risk factors and positive protective factors—associated with cognitive performance in later life.

## METHODS

### Study design, setting and population

A community-based, cross-sectional study was conducted in urban settlements in the Southwest District of Delhi. This district has three sub-divisions; there are three *talukas*, 41 villages, and 23 towns in it. The study duration was six months, from January to June 2023, and the study involved elderly individuals over 60 years old living in urban settlements of the Southwest District of Delhi.

### Sampling design

A multistage sampling approach was adopted for this study. At the first stage, systematic listings of all housing societies within the selected wards were prepared. Societies were then selected using convenience sampling. The president of each selected society was contacted, and a detailed house listing was carried out for sampling purposes. In the second stage, wards were selected from each block through convenience sampling. In the third stage, households within the selected wards were chosen using consecutive sampling. A pilot survey was conducted before data collection to estimate the proportion of households without elderly residents (over the age of 60), ensuring that the sample size would be adequate. The required sample size was calculated using N Master software, with a 10% allowance for non-response. The estimated sample size was 288, and data collection was completed for 290 participants.

### Inclusion and exclusion criteria

Participants aged 60 years or older, residing in the urban settlements of Southwest Delhi for at least six months and able to provide informed consent, were included in the study. Individuals were excluded if they had a diagnosed neurological disorder (such as stroke, dementia, or Parkinson's disease), severe psychiatric illness, learning or intellectual disabilities, uncorrected sensory impairments that prevented interviewing, were critically ill or hospitalized at the time of data collection, or were unable to communicate or respond reliably during the assessment.

### Exposure assessment

A total of 290 participants aged above 60 years were recruited for the study. Prior written permission was obtained from each of the selected societies. Households were chosen through convenience sampling, and eligible participants who met the study criteria were enrolled after obtaining their informed consent. Data collection was conducted using paper-based standardized and structured questionnaires. Interviews were conducted to gain information regarding socio-demographic characteristics, psychosocial characteristics, physical activity, and behavioral risk factors such as alcohol consumption and smoking were collected. Height was measured using a portable stadiometer. Weight was measured using a digital weighing scale. As the interview contained some sensitive questions, the data were collected in privacy.

The following are socio-demographic variables. Three age groups were established: 61–70 years, 71–80 years, and 81 years and older. Sex was divided into male and female categories. Technical/skilled worker, clerk, shopkeeper, semi-skilled worker, unskilled worker, and unemployed were categorized for previous occupations. There are four categories of educational status: primary/middle school, illiterate/no formal education, and high school/graduate/diploma. The terms "married," "not married," "single," "divorced," "separated," and "widowed" were used to denote marital status. Families were classified as nuclear, joint, or living alone. Additional categories included no children, two or less than two, three, or more children. Indicators of BMI include underweight, normal weight, overweight, and obese. Heart disorders, diabetes, and other medical conditions were classified as medical conditions. Utilization of medications is a binary variable. The following variables are shown as behavioral variables, such as alcohol, smoking, and physical exercise, and are all binary variables. Daily, weekly, and monthly physical activity frequency examples were provided. Physical activity was broken down into categories of at least 10 minutes, 10–30 minutes, 30–1 hour, and more than an hour. Past smokers and current smokers were two categories for smoking frequency. Alcohol use was divided into three categories: frequently, sporadically, and infrequently. According to illustrations, smoking and drinking can last 25, 26, and 50 years, respectively. Variables such as sleep, diet and relationship with family and friends were categorized as binary. The variable on mental activity was categorized into regularly, periodically and rarely.

### Outcome assessment

Interviews were conducted using standardized measurement scales known as BIMS. This is a standardized tool designed to assess cognitive function, particularly memory and orientation, in older adults. It is divided into three sections: instantaneous recollection, temporal orientation, and delayed recall. The instant recall segment requires participants to remember and repeat three unrelated words, whereas temporal orientation assesses their knowledge of the present year, month, and day of the week. After a brief pause, participants are asked to recall the first three words. The BIMS score runs from 0 to 15, with higher values indicating greater cognitive function, and thresholds are used to determine if an individual has intact, moderate, or severe cognitive impairment. The BIMS scale was used to assess cognitive impairment under three main categories. With this, a person's attention, orientation, and ability to register and recall were assessed. A total score of 13 to 15 meant intact cognition, 08 to 12 meant moderate impairment and less than 7 was severe cognitive impairment^21^.

A coefficient of 0.8600 suggests a relatively high level of internal consistency, indicating that the items in the scale are closely related to each other and are measuring the same underlying construct. This suggests that the scale is reliable for measuring the intended construct, with a good level of consistency among its items.

### Statistical analysis

The data were entered in Census and survey processing system (CS Pro version 7.0) and was analyzed using STATA software, version 15.1. The statistical analysis for this study included many critical procedures to thoroughly investigate predictive factors and their correlations with the outcome variable. All statistical tests were two-tailed, and statistical significance was defined at α=0.05. To reduce dimensionality, identify key explanatory variables, and minimize overfitting, we conducted Principal Component Analysis (PCA) on standardized continuous predictors. The proportional odds assumption underlying the ordinal logistic regression was assessed using the Brant test, which indicated no significant violation of the assumption. Components with Eigenvalues ≥1 and explaining up to 80% of cumulative variance were retained. Variables showing absolute loadings ≥0.30 on the first eight components were selected for inclusion. Where overlapping constructs existed — such as continuous variables and their categorized forms — we retained the form with stronger theoretical relevance or higher component loading. This ensured a parsimonious selection of predictors, balancing data-driven insights with conceptual validity^22^; where a_ij_ is the loadings of variables X_j_ on the i-th principal component.


$${\text{PC}}_{\text{i}}={\text{a}}_{\text{i1}}{\text{X}}_{\text{1}}+{\text{a}}_{\text{i2}}{\text{X}}_{\text{2}}+\ldots +{\text{a}}_{\text{ip}}{\text{X}}_{\text{p}}$$

We further assessed multicollinearity among selected variables using the Variance Inflation Factor (VIF), adopting a threshold of VIF > 5 to indicate potential concern. Variables exceeding this threshold were either excluded or consolidated based on theoretical importance and contribution to model stability^23^. R^2^_i_ is the coefficient of determination for predictor i when regressed on all other predictors.


$${\text{VIF}}_{\text{i}}=\frac{1}{\left(1-R_{\text{i}}^{2}\right)}$$

Subsequently, we used ordinal logistic regression to examine the impact of several factors (such as socioeconomic status, physical health, and mental health disorders) on cognitive impairment, which was classified as intact, mild to moderate, or severe. This analysis uses Stata's "ologit" command to estimate odds ratios and 95% confidence intervals^24^. Here, α_k_ is the intercept for category threshold k, and β_p_ are coefficients for predictors X_p_.


$$\log(\frac{\text{P}(\text{Y}>\text{k})}{\text{P}(\text{Y}\leq \text{k})})={\text{α}}_{\text{k}}-{\text{β}}_{\text{1}}{\text{X}}_{1}-{\text{β}}_{2}{\text{X}}_{2}-\ldots -{\text{β}}_{\text{p}}{\text{X}}_{\text{p}}$$

Following the ordinal logistic regression analysis, we conducted an exploratory K-means clustering to identify potential subgroups of participants based on patterns in their cognitive domain scores^25^. Using repetition of words, temporal orientation, and recall scores from the BIMS scale as continuous indicators, K-means clustering partitioned participants into mutually exclusive groups by minimizing within-cluster variance based on Euclidean distance. The number of clusters (k = 2) was chosen for its interpretability and meaningful separation of cognitive profiles^26^. Each participant was assigned to the cluster that best reflected their cognitive performance pattern. Cluster-specific distributions of cognitive domain scores were summarized and visualized using boxplots, enabling comparison of cognitive profiles between the identified groups. This clustering analysis complemented the regression findings by offering an unsupervised perspective on potential cognitive subtypes within the study population—minimizing the total within-cluster sum of squares (WCSS), where WCSS is the within-cluster sum of squares, μ_i_ is the centroid of cluster i, and x are the data points assigned to cluster i.


$$\text{WCSS}=\sum_{\text{i}=1}\text{k}\sum_{\text{x}}\in {\text{C}}_{\text{i}}\|\text{x}-{\text{μ}}_{\text{i}}{\|}^{2}$$

### Ethics approval

The ethical approval for this study was obtained from the Institutional Ethics Committee at IIPH-Delhi (Approval No.: IIPHD_IEC_MSC CR_S_8_2023), and all study procedures adhered to the approved protocol. All the participants were informed of the purpose of the study in simple and local language (Hindi), using the Participant Information Sheet (PIS). Participants were made aware of the voluntary nature of their participation, understanding that they could choose to leave the study or refuse to complete the interview at any point. Prior informed consent was obtained from respective participants in Hindi and English before collecting any information from the participants. All information collected from the participants were kept confidential and will not be shared with anybody outside the research team. The BIMS and other outcome measures were administered in English by trained interviewers to ensure linguistic and cultural appropriateness, and standardized administration procedures were followed. All the procedures established for this study adhered to the 1964 Declaration of Helsinki and were in accordance with the applicable regulatory requirements.

## RESULTS

Table 1 depicts the socio-demographic characteristics of the study participants. Among the 290 elderly participants included in this community-based study from urban Delhi, 49.31% were aged 60–70 years, and 44.48% were female. The majority were either skilled workers (51.03%) or unemployed (24.48%), and 75.86% had completed high school or higher education. Most participants resided in nuclear families (73.79%), and were currently married (77.24%). Approximately one-third (34.14%) had three or more children. Regarding nutritional status, 37.24% were of normal weight, while 21.03% were classified as obese. The prevalence of chronic conditions was notable, with 62.76% reporting hypertension or diabetes, and 18.28% reporting heart disease. Ongoing medication use was reported by 95.17% of participants.

**Table 1:** Socio-demographic, health, behavioural, and lifestyle characteristics of elderly participants in urban settlements of Delhi.

Characteristics		n (%) n=290
Age (mean)		Mean=71.62
Age (years)		
	60 to 70	143 (49.31)
	71 to 80	108 (37.24)
	81 and above	39 (13.45)
Sex		
	Male	151 (55.52)
	Female	129 (44.48)
Previous occupation		
	Technician/Skilled worker	148 (51.03)
	Semi-skilled worker	51 (17.59)
	Unskilled worker	20 (6.90)
	Unemployed	71 (24.48)
Educational status		
	High school/Graduate/Diploma	220 (75.86)
	Primary/Middle school	28 (9.66)
	Illiterate/No formal education	42 (14.48)
Marital status		
	Married	224 (77.24)
	Not married/Single	6 (2.07)
	Divorced/Separated/Widowed	60 (20.69)
Type of family		
	Nuclear	214 (73.79)
	Joint	42 (14.48)
	Staying alone	34 (11.72)
Number of children		
	No child	17 (5.86)
	2 or less than 2	174 (60.00)
	3 or more	99 (34.14)
Body mass index		
	Underweight	11 (3.79)
	Normal weight	108 (37.24)
	Overweight	110 (37.93)
	Obese	61 (21.03)
Any medical condition		
	Hypertension/Diabetes mellitus	182 (62.76)
	Heart disease	53 (18.28)
	Others	55 (18.97)
Any ongoing medication		
	Yes	276 (95.17)
	No	14 (4.83)
Physical activity		
	Yes	144 (49.66)
	No	146 (50.34)
Frequency of physical activity		
	Daily	82 (28.28)
	Weekly	58 (20.00)
	Monthly	7 (2.41)
	No physical activity	143 (49.31)
Smoking/ever smoked		
	Yes	113 (38.97)
	No	177 (61.03)
Alcohol		
	Yes	131 (45.17)
	No	159 (54.83)
Frequency of smoking		
	Past smoker	64 (22.07)
	Current smoker	49 (16.90)
	Non-smoker	177 (61.03)
Frequency of alcohol		
	Regularly	32 (11.03)
	Occasionally	77 (26.55)
	Rarely	22 (7.59)
	Does not drink alcohol	159 (54.83)
Duration of smoking (years)		
	<25	6 (2.07)
	26–50	61 (21.03)
	51 or more	46 (15.86)
	Non-smoker	177 (61.03)
Duration of alcohol (years)		
	<25	13 (4.48)
	26–50	86 (29.66)
	51 or more	32 (11.03)
	Does not drink alcohol	159 (54.83)
Dietary intake		
	Inadequate	118 (40.69)
	Adequate	172 (59.21)
Sleeping pattern		
	Improper	182 (62.76)
	Proper	108 (37.24)
Mental activity		
	Regularly	135 (46.45)
	Periodically	80 (27.59)
	Rarely	75 (25.86)
Social involvement		
	No	126 (43.45)
	Yes	164 (56.55)
Relationship with friends and family		
	No	151 (52.07)
	Yes	139 (47.93)

Table 2 shows significant bivariate associations between cognitive status and multiple socio-demographic, health, behavioral, and lifestyle factors among elderly participants in urban Delhi. Age and education emerged as strong correlates; 34.5% of those aged 60–70 years and 43.8% of those with high school education or above had intact cognition, while severe impairment was highest among those aged ≥81 years (5.9%) and the illiterate (5.5%) (p<0.001). Women had a higher proportion of both intact and severely impaired cognition compared to men (p<0.001). Skilled occupation, being married, and living in nuclear families were associated with better cognitive outcomes (p<0.001).

**Table 2:** Bivariate associations between socio-demographic, health, behavioural, and lifestyle characteristics and cognitive status among elderly residents in urban settlements of Delhi.

Characteristics		Degree of cognition (n=290)			p-value
		Intact n (%) n=153	Mild to moderate n (%) n=108	Severe n (%) n=29	
Age (years)					
	60 to 70	100 (34.48)	40 (13.79)	3 (1.03)	<0.001
	71 to 80	48 (16.55)	51 (17.59)	9 (3.10)	
	81 and above	5 (1.72)	17 (5.86)	17 (5.86)	
Sex					
	Male	71 (24.48)	76 (26.21)	14 (4.83)	<0.001
	Female	82 (28.28)	32 (11.03)	29 (5.17)	
Previous occupation					
	Technician/skilled worker	77 (26.55)	64 (22.97)	7 (2.41)	<0.001
	Semi-skilled worker	33 (11.38)	14 (4.83)	4 (1.38)	
	Unskilled worker	5 (1.72)	12 (4.14)	3 (1.03)	
	Unemployed	38 (13.10)	18 (6.21)	15 (5.17)	
Educational status					
	High school/Graduate/Diploma	127 (43.79)	82 (28.28)	11 (3.79)	<0.001
	Primary/Middle school	13 (4.48)	13 (4.48)	2 (0.69)	
	Illiterate/No formal education	13 (4.48)	13 (4.48)	16 (5.52)	
Marital status					
	Married	129 (44.48)	83 (28.62)	12 (4.14)	<0.001
	Not married/Single	3 (1.03)	3 (1.03)	0	
	Divorced/Separated/Widowed	21 (7.24)	22 (7.59)	17 (5.86)	
Type of family					
	Nuclear	127 (43.79)	69 (23.79)	18 (6.21)	<0.001
	Joint	14 (4.83)	23 (7.93)	5 (1.72)	
	Staying alone	12 (4.14)	16 (5.52)	6 (2.07)	
Number of children					
	No child	8 (2.76)	8 (2.76)	1 (0.34)	0.864
	2 or less than 2	90 (31.03)	66 (22.76)	18 (6.21)	
	3 or more	55 (18.97)	34 (11.72)	10 (3.43)	
Body mass index					
	Underweight	1 (0.34)	4 (1.38)	6 (2.07)	<0.001
	Normal weight	46 (15.86)	48 (16.55)	14 (4.83)	
	Overweight	67 (23.10)	38 (13.10)	5 (1.72)	
	Obese	39 (13.45)	18 (6.21)	4 (1.38)	
Any medical condition					
	Hypertension/Diabetes mellitus	107 (36.90)	62 (21.38)	13 (4.48)	<0.001
	Heart disease	11 (3.79)	29 (10.00)	13 (4.48)	
	Others	35 (12.07)	17 (5.86)	3 (1.03)	
Any ongoing medication					
	Yes	140 (48.28)	107 (36.90)	29 (10.00)	0.009
	No	13 (4.48)	1 (0.34)	0	
Behavioural and lifestyle factors					
Physical activity					
	Yes	97 (33.45)	41 (14.14)	6 (2.07)	<0.001
	No	56 (19.31)	67 (23.10)	23 (7.93)	
Frequency of physical activity					
	Daily	66 (22.76)	15 (5.17)	1 (0.34)	<0.001
	Weekly	29 (10.00)	27 (9.31)	2 (0.69)	
	Monthly	2 (0.69)	2 (0.69)	3 (1.03)	
	No physical activity	56 (19.31)	64 (22.07)	23 (7.93)	
Smoking/ever smoked					
	Yes	36 (12.41)	64 (22.07)	13 (4.48)	<0.001
	No	117 (40.34)	44 (15.17)	16 (5.52)	
Alcohol					
	Yes	50 (17.24)	69 (23.79)	12 (4.14)	<0.001
	No	103 (35.52)	39 (13.45)	17 (5.86)	
Frequency of smoking					
	Past smoker	17 (5.86)	37 (12.76)	10 (3.45)	<0.001
	Current smoker	19 (6.55)	27 (9.31)	3 (1.03)	
	Non-smoker	153 (52.76)	44 (15.17)	16 (5.52)	
Frequency of alcohol					
	Regularly	9 (3.10)	19 (6.55)	4 (1.38)	<0.001
	Occasionally	29 (10.00)	43 (14.83)	5 (1.72)	
	Rarely	12 (4.14)	7 (2.41)	3 (1.03)	
	Does not drink alcohol	103 (35.52)	39 (13.45)	17 (5.86)	
Duration of smoking (years)					
	<25	2 (0.69)	4 (1.38)	0	<0.001
	26–50	24 (8.28)	32 (11.03)	5 (1.72)	
	51 or more	10 (3.45)	28 (9.66)	8 (2.76)	
	Non-smoker	117 (52.76)	44 (15.17)	16 (5.52)	
Duration of alcohol (years)					
	<25	8 (2.76)	5 (1.72)	0	<0.001
	26–50	36 (12.41)	43 (14.83)	7 (2.41)	
	51 or more	6 (2.07)	21 (7.24)	5 (1.72)	
	Does not drink alcohol	103 (35.52)	39 (13.45)	17 (5.86)	
Dietary intake					
	Inadequate	11 (3.79)	81 (27.93)	26 (8.97)	<0.001
	Adequate	142 (48.97)	27 (9.31)	3 (1.03)	
Sleeping pattern					
	Improper	65 (22.41)	88 (30.34)	29 (10.00)	<0.001
	Proper	88 (30.34)	20 (6.90)	0	
Mental activity					
	Regularly	125 (43.45)	8 (2.76)	1 (0.34)	<0.001
	Periodically	19 (6.55)	52 (17.93)	9 (3.10)	
	Rarely	8 (2.76)	48 (16.55)	19 (6.55)	
Social involvement					
	No	8 (2.76)	90 (31.03)	28 (9.66)	<0.001
	Yes	145 (50.00)	18 (6.21)	1 (0.34)	
Relationship with friends and family					
	No	44 (15.17)	83 (28.62)	24 (8.28)	<0.001
	Yes	109 (37.59)	25 (8.62)	5 (1.72)	

Health-related factors such as BMI, chronic conditions, and medication use were also associated with cognition. Underweight individuals and those with heart disease had higher rates of severe impairment, while overweight and normotensive individuals were more likely to have intact cognition (p<0.001). Physical activity showed a strong protective association — 33.5% of active individuals had intact cognition, compared to 7.9% of inactive participants who were severely impaired. Substance use (smoking and alcohol), inadequate diet, poor sleep, lack of mental activity, social isolation, and weak relationships were all significantly linked with cognitive impairment (p<0.001).

**Figure 1:**
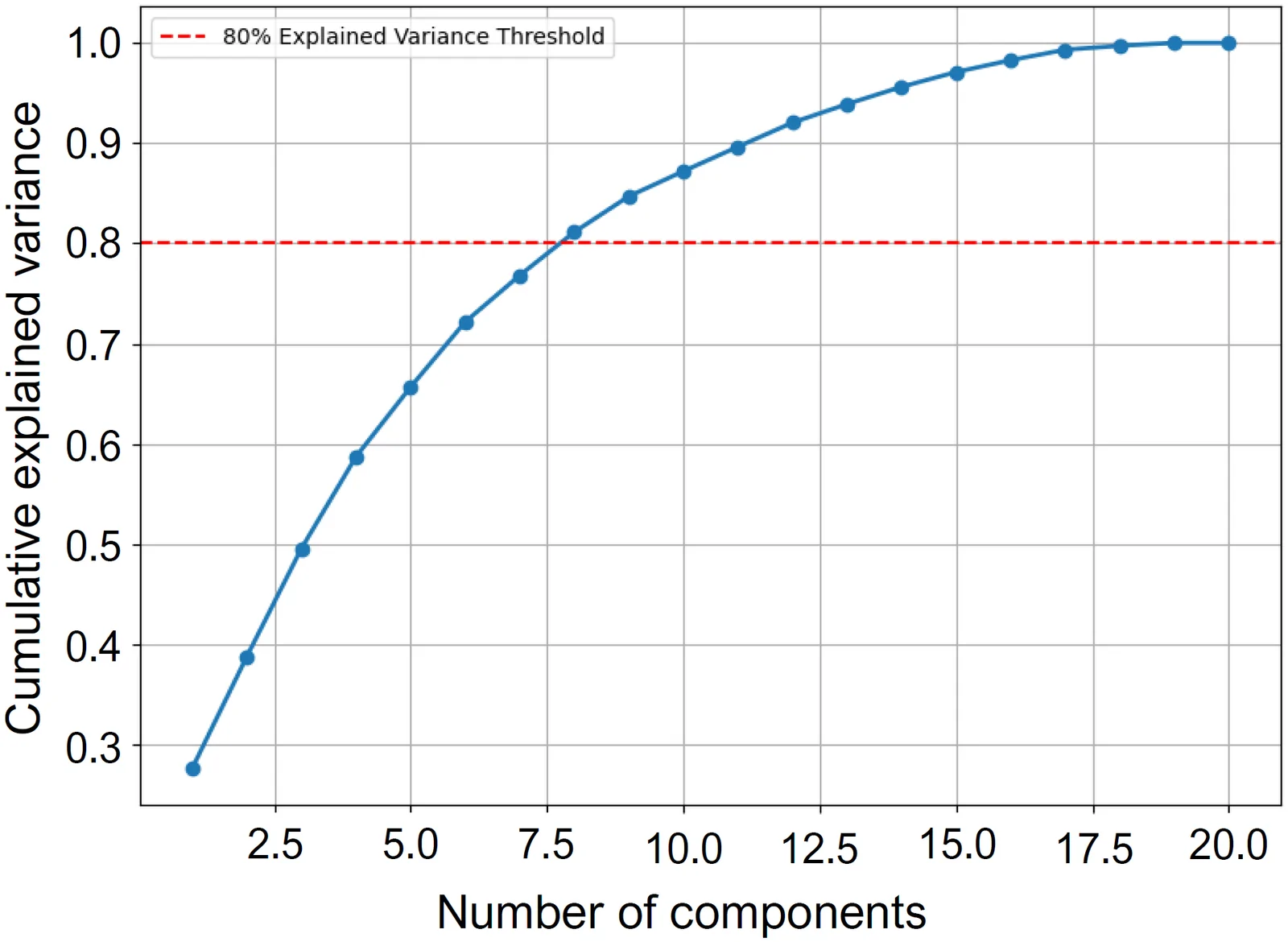
Cumulative explained variance by principal components showing the retention of the first eight components capturing 80% of total variance.

PCA identified eight principal components that together explained approximately 80% of the total variance in the predictor variables. The first component (PC1) primarily reflected lifestyle-related factors, with high positive loadings from smoking duration, alcohol consumption, and age. The second component (PC2) was characterized by strong loadings from BMI and BMI category, alongside alcohol-related variables, indicating a cluster of metabolic and substance use factors. The third component (PC3) was dominated by education and occupation, capturing socioeconomic status. The fourth component (PC4) showed high negative loadings from medical conditions and social involvement, highlighting a potential health and social support dimension. Other components captured distinct combinations of family structure, sleep, mental activity, and social relationships. These components, selected based on their contribution to the explained variance and variable loadings, informed the choice of variables included in the ordinal regression model, ensuring representation of the main latent dimensions underlying the dataset (Figure 1).

The results of the multivariable ordinal logistic regression analysis presented in Table 3, adjusted for potential confounders, reveal distinct associations between socio-demographic, health-related, and behavioral variables with levels of cognitive impairment among elderly participants. Regarding socio-demographic factors, participants aged over 80 years showed non significantly lower odds of cognitive impairment (Adjusted Odds Ratio – AOR 0.517; 95% confidence interval – 95%CI 0.101–2.655) compared to those aged 60–70 years. Lower educational attainment was strongly associated with cognitive impairment; individuals with no formal education had over 75% lower odds of preserved cognition (AOR 0.044; 95%CI 0.007–0.296) than those with high school or higher education. However, no significant association was observed with marital status, type of family, or the number of children. Among health-related variables, BMI emerged as a critical factor. Those with normal BMI (AOR 10.43; 95%CI 1.746–83.072) and overweight status (AOR 8.06; 95%CI 1.863–51.703) had significantly higher odds of better cognitive outcomes compared to underweight participants. The presence of heart disease was associated with lower odds of cognitive impairment (AOR 0.704; 95%CI 0.219–2.261), although not statistically significant. Ongoing medication did not exhibit a significant association.

**Table 3:** Multivariable ordinal logistic regression of factors associated with cognitive impairment among elderly participants in Urban Delhi.

Characteristics		Unadjusted Odds Ratio (95%CI)	p-value	Adjusted Odds Ratio (95%CI)	p-value
Age (years)					
	60–70	Reference		Reference	
	70–80 yeas	0.339 (0.203–0.567)	<0.001	2.05 (0.617–6.811)	0.241
	81 and above	0.044 (0.019–0.099)	<0.001	0.517 (0.101–2.655)	0.429
Previous occupation					
	Technician/skilled worker	Reference		Reference	
	Semi-skilled worker	1.507 (0.789–2.876)	0.214	0.243 (0.059–0.999)	0.05
	Unskilled worker	0.370 (0.157–0.872)	0.023	0.707 (0.103–4.856)	0.724
	Unemployed	0.774 (0.440–1.357)	0.371	0.35 (0.061–2.021)	0.241
Educational status					
	High school/Graduate/Diploma	Reference		Reference	
	Primary/Middle school	0.657 (0.311–1.386)	0.270	0.553 (0.095–3.232)	0.511
	Illiterate/No formal education	0.183 (0.091–0.368)	<0.001	0.047 (0.007–0.296)	0.001
Number of children					
	No child	Reference		Reference	
	2 or more	1.073 (0.422–2.726)	0.881	0.656 (0.109–3.941)	0.645
	3 or more	1.229 (0.467–3.234)	0.676	0.594 (0.092–3.845)	0.584
Body mass index					
	Underweight	Reference		Reference	
	Normal weight	8.401 (2.420–29.167)	0.001	12.043 (1.746–83.072)	0.012
	Overweight	18.437 (5.217–65.154)	<0.001	6.681 (0.863–51.703)	0.069
	Obese	20.109 (5.412–74.719)	<0.001	4.099 (0.493–34.102)	0.192
Any medical condition					
	Hypertension/Diabetes Mellitus	Reference		Reference	
	Heart disease	0.202 (0.109–0.373)	<0.001	0.704 (0.219–2.261)	0.556
	Others	1.237 (0.669–2.289)	0.497	0.128 (0.032–0.513)	0.004
Any ongoing medication					
	Yes	0.078 (0.010–0.606)	<0.015	0.059 (0.001–3.114)	0.162
	No	Reference		Reference	
Physical activity					
	Yes	Reference		Reference	
	No	0.291 (0.182–0.466)	<0.001	1.433 (0.488–4.205)	0.513
Smoking					
	Yes	0.300 (0.188–0.479)	<0.001	5.568 (1.187–26.127)	0.029
	No	Reference		Reference	
Alcohol					
	Yes	Reference		Reference	
	No	2.409 (1.525–3.804)	<0.001	4.027 (0.902–17.975)	0.068
Dietary intake					
	Inadequate	Reference		Reference	
	Adequate	39.376 (19.937–77.768)	<0.001	10.445 (2.844–38.367)	<0.001
Sleeping pattern					
	Improper	Reference		Reference	
	Proper	8.412 (4.762–14.860)	<0.001	7.224 (1.865–27.983)	0.004
Mental activity					
	Regularly	Reference		Reference	
	Periodically	0.022 (0.009–0.052)	<0.001	0.03 (.006–0.166)	<0.001
	Rarely	0.008 (0.003–0.021)	<0.001	0.028 (0.005–0.168)	<0.001
Social involvement					
	No	Reference		Reference	
	Yes	104.956 (45.835–240,333)	<0.001	67.517 (15.684–290.653)	<0.001
Relationship with friends and family					
	No	Reference		Reference	
	Yes	0.361 (4.950–14.121)	<0.001	4.863 (1.642–14.404)	0.004

Notes: Model summary: Pseudo R-squared=0.718; Number of observations=290; Model χ^2^=389.340.

Behavioral factors demonstrated strong relationships with cognitive outcomes. Physically active individuals had markedly reduced odds of cognitive impairment (AOR 1.433; 95%CI 0.438–4.205). Smoking and alcohol use were also significant; smokers (AOR 5.568; 95%CI 1.187–26.127) and alcohol consumers (AOR 4.902; 95%CI 1.797–17.975) showed higher odds of impairment. Poor dietary intake was inversely associated with cognitive function (AOR 0.053; 95%CI 0.005–0.616), while improper sleep patterns were strongly linked with impairment (AOR 10.45; 95%CI 2.844–38.567). Participants engaging in regular mental activity (AOR 0.007; 95%CI 0.000–0.166) and those with social involvement (AOR 0.003; 95%CI 0.001–0.063) had significantly lower odds of cognitive impairment. Notably, maintaining positive relationships with family and friends was associated with a four-fold increase in the likelihood of better cognitive status (AOR 4.263; 95%CI 1.642–14.404) (Table 3).

Model diagnostics indicated a good fit with a pseudo-R-squared of 0.718, a highly significant chi-square statistic (χ^2^=389.340, p<0.001), and acceptable Akaike Information Criterion – AIC (205.233) and Bayesian Information Criterion – BIC (300.650) values, confirming the robustness of the model in explaining cognitive outcomes (Table 3).

**Figure 2:**
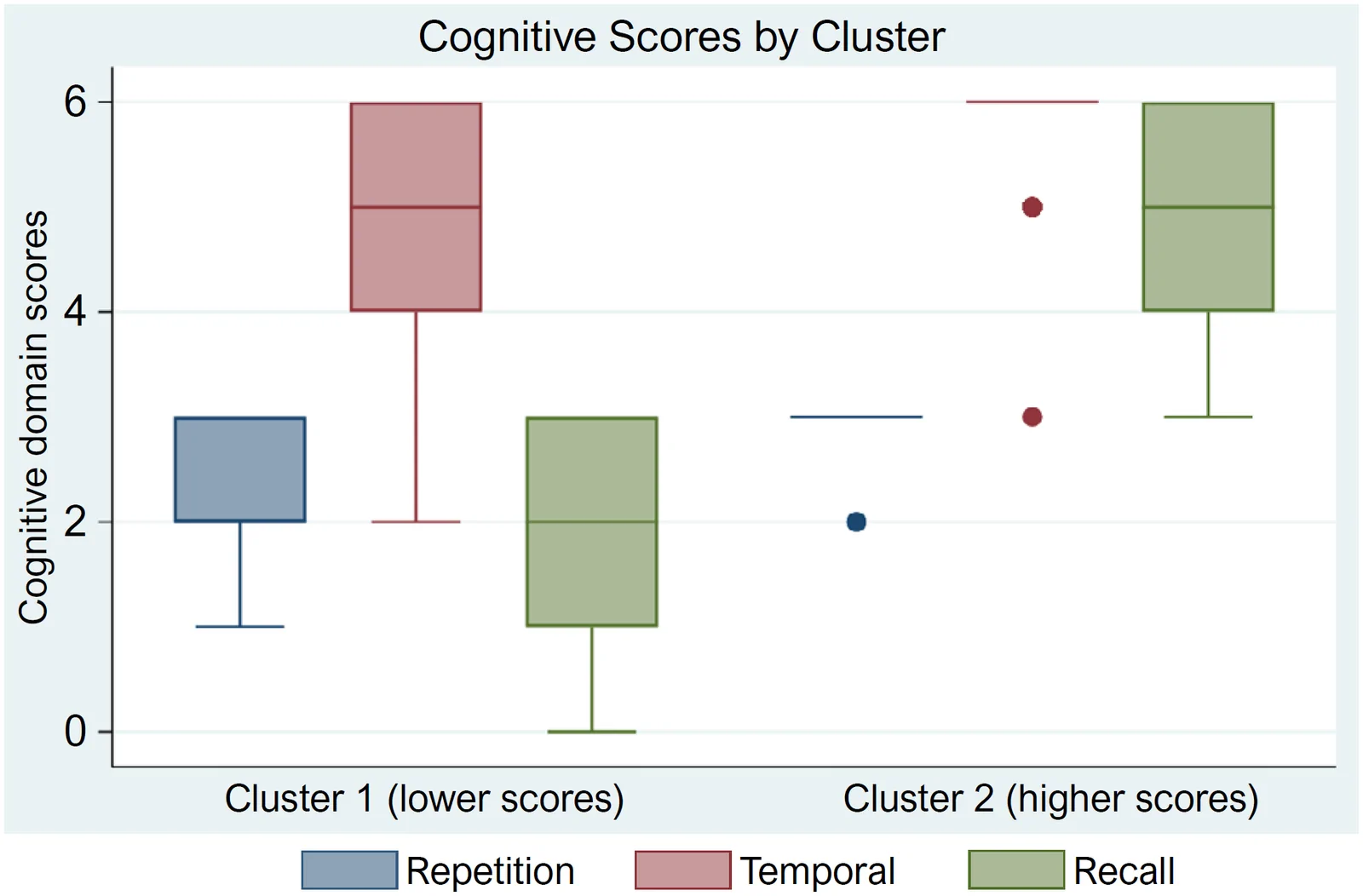
Boxplot of repetition, temporal orientation, and recall scores by K-means derived clusters.

K-means clustering analysis identified two distinct participant groups based on their cognitive domain scores in repetition, temporal orientation, and recall. As shown in Figure 2, participants classified under "Cluster 1 (Lower Scores)" exhibited lower median scores with wider variability across all cognitive domains — most notably in recall — suggesting a pattern indicative of cognitive impairment. In contrast, those in "Cluster 2 (Higher Scores)" demonstrated higher median scores with narrower interquartile ranges, reflecting comparatively better cognitive performance.

The descriptive summary in Table 4 supported these patterns, with Cluster 1 participants showing lower mean scores in repetition (2.29), temporal orientation (4.98), and recall (1.67), compared to Cluster 2, where the mean scores were higher — 2.99, 5.92, and 4.92 respectively. These results suggest that Cluster 1 may correspond to individuals with greater cognitive deficits, while Cluster 2 represents those with relatively intact cognitive abilities.

**Table 4:** Comparison of mean cognitive domain scores across K-means derived clusters.

Cluster	Mean repetition score	Mean temporal orientation score	Mean recall score
Cluster 1 (lower score)	2.29	4.98	1.67
Cluster 2 (higher score)	2.99	5.92	4.92

## DISCUSSION

In the current study, 290 elderly individuals were used as a sample, and the relationship between demographics and behavioral characteristics and the severity of cognitive impairment was investigated. Severe (10.00%), mild to moderate (37.24%), and intact cognition (52.8%) were the three levels of cognitive impairment prevalence. Our findings demonstrated that cognitive impairment was significantly associated with advancing age, lower educational attainment, underweight status, presence of hypertension or diabetes, lower levels of physical activity, smoking, alcohol use, inadequate dietary intake, irregular sleep patterns, low engagement in mental activities, limited social involvement, and weaker family and social relationships. Conversely, maintaining a healthy body weight, engaging in regular physical and mental activities, consuming an adequate diet, maintaining proper sleep patterns, and fostering strong social ties were associated with better cognitive function.

This study shows that a decrease in cognitive impairment correlates strongly with an increase in age. This is consistent with the results of a longitudinal study conducted by Overton et al. that determined the prevalence and incidence of moderate cognitive impairment with increasing age^27^. In our study, living alone was associated with compromised cognition in the bivariate analysis; however, this association did not persist after adjustment for other factors. Nonetheless, this finding resonates with a Spanish longitudinal study by Lara et al., which found that loneliness and social isolation were both linked to decreased cognitive function over a 3-year follow-up period^28^.

This study's findings that chronic illnesses are significantly linked to poor cognition coincide with the results of a recent longitudinal Chinese study by Jiang et al., which found a strong association between chronic illnesses and depressive symptoms in Chinese older adults^29^. This relationship was found to be mediated by cognitive impairment. Additionally, while previous studies reported no differences between urban and rural areas in how chronic diseases affect depressive symptoms, our study was conducted exclusively in urban settlements, and thus, such comparisons could not be assessed^29-31^. In our study, medication use among the elderly was significantly associated with cognitive impairment in the bivariate analysis; however, this association did not remain significant after adjustment. This is in line with findings by Fox et al.^32^, who established that the use of medications with anticholinergic activity increases the cumulative risk of cognitive impairment and mortality^32,33^.

This study supports the findings from this trial by Cordes et al.^34^, and provides evidence for multicomponent exercises that specifically focus on cognitive-motor approaches in the maintenance of mental and physical functioning. Behavioral determinants like physical activity are also a major factor for the cognitive functioning of the individual. Additionally, it will support promoting older individuals’ active participation in social life^34,35^. According to the study's findings, smoking reduces an individual's likelihood of having intact cognition compared to not smoking, which is consistent with a cross-sectional study Muhammad et al. conducted in India, which found a significant link between smoking, drinking alcohol, and using tobacco products and impaired cognition^36^. Similarly, the participants who consume alcohol have lower odds of intact cognition, which is not in alignment with the findings of the studies by Yen et al. and Lao et al. These latter studies indicated that participants between the ages of 60 and 69 who drank moderately to heavily had a lower risk of cognitive impairment, and established non-linear relationship between cognition and alcohol consumption^37,38^.

Inadequate dietary intake emerged as a significant predictor of cognitive impairment, highlighting the critical role of nutrition in maintaining cognitive health. This aligns with findings from Rivan et al., who reported that unhealthy dietary patterns substantially increase the risk of mild cognitive impairment and dementia^39^. Similarly, a large survey conducted in the United States demonstrated that higher dietary fiber intake is associated with better cognitive performance among older adults^40^. Irregular sleep patterns were strongly linked with cognitive impairment, emphasizing the growing recognition of sleep disturbance as a key factor in cognitive aging. This is supported by longitudinal findings where short and highly variable sleep were associated with increased cognitive decline^41^. Neurobiological evidence also indicates that sleep disruption can accelerate amyloid accumulation and neurodegeneration in aging brains^42^.

Moreover, regular engagement in mentally stimulating activities and active social involvement were each associated with better cognitive outcomes, reinforcing extensive literature on cognitive and social engagement as protective factors in aging^43^. These behaviours contribute to cognitive reserve and resilience against decline^44^.

Finally, our cluster analysis delineated two distinct cognitive profiles: one characterized by lower cognitive function and higher exposure to adverse lifestyle and health risk factors, and the other by better cognitive performance linked to protective behaviors and favorable socio-demographic characteristics. These subgroup patterns underscore the heterogeneity of cognitive aging and confirm the importance of combined socio-demographic, health, and behavioral strategies in designing interventions. These domain-wise cognitive profiles have practical implications for public health and clinical practice. Identifying older adults with deficits in attention, memory, or orientation may help guide early risk stratification and targeted interventions within community settings. Evidence from multidomain behavioral trials such as the FINGER study^45^, APPLE-Tree^46^, and DREAMS-START^47^ demonstrates that structured lifestyle support, cognitive stimulation, and social engagement can slow cognitive decline and improve wellbeing in older adults. Additionally, the Lancet Commission on dementia prevention highlights physical activity, nutrition, smoking cessation, sleep hygiene, and social connectedness as modifiable targets with substantial preventive potential^48^. Integrating such intervention strategies into primary care, urban health missions, and elderly welfare programmes in India could therefore enhance early detection, rehabilitation planning, and resource allocation, strengthening existing epidemiological responses to population ageing.

### Strengths of the study

The study uses a community-based methodology to ensure a more inclusive representation of the senior population living in metropolitan Delhi. This design decision improves the findings’ capacity to be applied to a larger population, which raises the study's relevance and usefulness. Additionally, the research employs a thorough methodology by looking into risk factors for both behavioral and other determinants along with cognitive impairment. This comprehensive evaluation enables a detailed knowledge of the numerous elements affecting older people›s cognition. The study also gains from a sizable sample size, which improves the statistical power and dependability of the outcomes. The involvement of a sizable number of participants boosts the study's credibility by instilling faith in the reliability of the results.

### Limitations of the study

The cross-sectional design limits causal inference, as it captures associations at a single point in time. Longitudinal studies are needed to clarify temporal relationships. Moreover, the consecutive sampling strategy at the society, ward, and household levels may introduce selection bias, potentially affecting sample representativeness. Reliance on self-reported data introduces the risk of recall bias and misreporting, particularly regarding behavioral factors and medical conditions. The absence of a control group restricts comparisons with the general population. Additionally, potential confounders may not have been fully accounted for due to resource constraints, raising the possibility of residual confounding. Although the BIMS has been previously administered in English among older adults in India and can be used without altering the meaning or components of the tool, the absence of formal cultural validation or India-specific normative cut-offs remains a limitation^49^. Additionally, the BIMS may be influenced by participants’ educational attainment and literacy levels; there is a risk of over- or underestimating cognitive impairment among individuals with lower formal education. Future research should consider cognitive measures that are less dependent on educational background. Finally, the findings are based on an urban, elderly population in Delhi and may not be generalizable to other settings or populations with different sociocultural contexts.

From a statistical perspective, several considerations warrant attention. PCA was used for dimensionality reduction and variable selection within the same dataset subsequently used for regression modelling, which may introduce data-driven selection bias and increase the risk of overfitting. The ordinal logistic regression model applied in this study relies on the proportional odds assumption across outcome categories; this assumption was formally assessed using the Brant test and showed no evidence of violation, supporting the appropriateness of the model and the validity of the estimated effect sizes. The K-means clustering analysis was exploratory in nature and inherently sensitive to variable scaling and cluster specification; accordingly, the identified cognitive profiles should be interpreted as descriptive patterns rather than definitive or causal subtypes. Furthermore, the relatively small number of participants classified with severe cognitive impairment may have limited statistical power and contributed to wide confidence intervals for certain estimates. The high pseudo-R² observed reflects explanatory performance within the study sample and should therefore not be interpreted as predictive accuracy or external validity. In conclusion, this study highlights the multifactorial determinants of cognitive impairment among older adults in urban Delhi, with significant associations observed for socio-demographic characteristics, chronic health conditions, and behavioral factors. Modifiable lifestyle factors — such as smoking, alcohol use, physical inactivity, inadequate diet, irregular sleep, and limited mental and social engagement — were linked to cognitive outcomes. These findings suggest the need for targeted interventions focusing on risk reduction strategies, including smoking cessation, promotion of physical activity, nutritional support, and social participation programs. Integrating such measures within community health initiatives may contribute to preserving cognitive function and delaying cognitive decline in the elderly population. The study adds evidence to support preventive approaches in geriatric cognitive health and informs future research and policy efforts in this area.

## Data Availability

The data that support the findings of this study are available from the corresponding author upon reasonable request.
